# Consumer Preferences and Sensory Profile Related to the Physico-Chemical Properties and Texture of Different Maize Tortillas Types

**DOI:** 10.3390/foods8110533

**Published:** 2019-10-31

**Authors:** Mădălina Iuga, Víctor D. Ávila Akerberg, Tanya M. González Martínez, Silvia Mironeasa

**Affiliations:** 1 Faculty of Food Engineering, Stefan cel Mare University of Suceava, 13, Universităţii Street, C.P. 720229 Suceava, Romania; iugamada@yahoo.com; 2 Instituto de Ciencias Agropecuarias y Rurales, Universidad Autónoma del Estado de México, Campus Universitario “El Cerrillo”, A.P. 435, Toluca, Estado de México C.P. 50200, Mexico; tanyamgm@gmail.com

**Keywords:** maize tortilla, consumer behavior, sensory profile, texture, physico-chemical parameters

## Abstract

Maize tortilla is a basic food in Mexico, and, lately, the food industry has tried to make the manufacturing process easier by using instant flours and specialized machines. The purpose of this study was to investigate consumers’ behaviors related to tortillas and to evaluate the sensory, textural, and physico-chemical parameters of tortillas from the Tlazala region, Mexico. The sensory profile revealed that the artisanal ones had better parameters in terms of smell, taste, and appearance compared to the others. These results are consistent with consumers’ preferences for tortillas made of maize grain instead of industrial corn flour. The sensory parameters and the physico-chemical and texture profile parameters varied with the maize type and manufacturing process. Our findings showed that the artisanal hand-made ones were more nutritious, followed by those mechanically made using maize grain, and finally by those mechanically made from industrialized corn flour. The results of this study may help processors to better understand the parameters of their products and people’s preferences.

## 1. Introduction

Maize is one of the most cultivated crops in America, Europe, and Asia, being largely consumed in Latin America. Maize is a key element in the Mesoamerican diet; however, only in Mexico is it mainly consumed in the shape of “tortilla”—a flat 12 to 18 cm disc made of nixtamalized maize flour, cooked over a hot comal or skillet [1]. Maize tortilla is one of the most popular foods in Mexico, and it is strongly related to the Mexican identity and considered a cultural heritage. It presents very different organoleptic parameters among Mexican territories, with the average daily consumption per capita being 180 g in urban areas and 300 g in rural zones of the country [2]. The materials used to obtain maize-based products, the processes, and equipment influence the nutritional value via loss of components [3]. Nixtamalization, a word coming from the indigenous Nahuatl linguistic root nixtli—meaning ashes or lime—and tamalli, a maize dough, is an ancient treatment (since 400–500 a. c.) used for maize grains. It involves grains boiling in approximately 5% lime water (calcium hydroxide) which enhances maize quality by softening the pericarp of the seed while increasing protein quality and the availability of niacin and calcium [4]. This treatment also promotes flavor development and improves tortillas’ consistency, while reducing the effects of fumosin, aflatoxin, deoxynivalenol, and zearalenone, all major contaminants of maize [4]. 

Tortillas are unfermented flat maize breads, with a soft, flexible, and easy to fold and roll structure and of various colors, depending on the flour and maize source [5]. In Mexico, there are more than 52 species and more than 350 cultivars/colors per species; they are called natives and are cultivated in traditional peasant farming systems, such as the cornfield, as well as genetically improved maize hybrids cultivated in intensive production systems with less costs [6,7]. Today, most of the commercial maize products consumed in Mexico are obtained from industrially grown maize imported from the United States, but in the center of Mexico native maize is still consumed. The quality differences among the tortillas on the market appear due to the fact that some artisanal tortillerias incorporate industrial maize flour gradually [6,8]. The traditional method of tortilla making involves maize grain nixtamalization to obtain the nixtamalized maize dough; however, nowadays, this procedure has been replaced by industrial nixtamalized maize flour [9]. Maize tortillas are a good source of proteins, providing important caloric intake. Taking into consideration peoples from Africa and Latin America, maize consumption is between 15% and 56% [10]. The chemical and physical parameters of nixtamalized maize and flour tortillas are influenced by the grain’s properties. The conditions of the nixtamalization process and the milling method also play an important role [10,11]. 

In rural households, the perception of quality, which is considered as “a good tortilla”, is closely related to the artisanal process, which also includes cooking in a wood-burning stove and clay dish [12]. Consumers’ preferences regarding tortillas depend on the region of the country [13]. In urban areas, tortillas of industrial origin are more readily accepted, while in rural areas, artisanal manufacturing, still dominated by women, is preferred [14,15]. Nowadays, traditional tortillas handmade by women tend to be replaced by the use of electric machines and gas combustion [16].

People’s choices regarding tortilla products is important for producers, especially from the product quality enhancement point of view. In rural areas, traditional agricultural practices registered substantial changes, mainly due to the free-trade policies which caused an increase in imported crops [17]. A commercial-scale production of tortillas implies changes to the traditional processes which lead to products with different sensory parameters. Some studies revealed that tortilla purchase intent is influenced by appearance, textural properties, and taste [18]. Industrialized tortillas, sold in self-service stores and mechanized tortillerias, tend to be much thinner, become hard quickly, and have a slightly sweet taste of maize which is almost imperceptible, while a lime flavor predominates [6]. These industrial tortillas are made with flours from large business monopolies, with imported maize that, due to the fact of their practicality, accessibility, shelf-life, and price, displace the original meaning of freshly made tortillas with their market model. Food perception and choices are different between women and men as a result of distinct energy needs, depending on their activities. Men consume foods with a higher energy density, while women prefer diets rich in vegetables, fruits, and fibers [19]. Regarding eating styles, men take bigger food bites and eat faster than women. Different food preferences among the two groups are related to the response at stimuli, such as visual image, gustatory information, emotions, hormonal changes, and weight status [19]. 

Tortilla quality can be evaluated by many methods, including sensory and objective methods. Many studies have been conducted to evaluate tortillas from different maize sources [9,10,20,21,22]. The mechanical textural properties of tortillas can be evaluated by elongation tests with the results depending on the product freshness and flour composition [9]. The color properties of the final product depend on the nixtamalization process with the intensity being related to the content of carotenoids and flavonoids and to Maillard reactions [23]. Tortilla sensory evaluation can bring information about acceptance, appearance, smell, taste, and textural properties. Bejosano et al. [22] revealed that sensory parameters and textural parameters evaluated by subjective and objective methods changed with time and presented significant correlations. The color, odor, flavor, shelf-life, and textural properties of nixtamalized tortillas are strongly influenced by lime concentration [23].

The aim of this study was to investigate the preferences related to tortillas and to evaluate the sensory, texture, and physico-chemical parameters of three types of maize tortillas among consumers from a small rural mountain village, Tlazala, Municipality of Isidro Fabela, which has experienced a rapid urbanization process over the last two decades due to the fact of its proximity to Mexico City’s Metropolitan Area. To this purpose, a questionnaire was applied and tortillas from different markets in Tlazala and from different maize sources were evaluated by determining the sensory profiles, texture parameters, color, chemical composition, and water absorption indexes. To our knowledge, no study has been performed on the quality of tortilla products from Tlazala, Mexico. Furthermore, this study focused on not only consumers’ behavior and sensory perceptions, but also on product parameters and their interactions.

## 2. Materials and Methods 

### 2.1. Consumers Preferences

Consumers’ behavior were evaluated by applying a questionnaire to 60 randomly selected tortilla consumers out of approximately 2000 habitants, of which 30 were women and 30 men, in Tlazala village, Mexico. People on the street in Tlazala’s center were asked to complete the questionnaire by choosing the answer from a given list. The collection period was between 25 January and 10 March 2019, and the working language was Spanish. The participants’ ages varied between 14 and 75 years old and they had different jobs. The questionnaire was divided into two sections: the first one regarding purchases and preferences and the second regarding purchase decision factors related to tortillas (Table 1). The hypothesis was that tortilla consumers’ preferences and purchase decisions depend on gender.

### 2.2. Materials

Eight tortillas samples were acquired from markets in the Tlazala region, Mexico. Six samples were obtained from specialized shops called “tortillerías” where tortillas are mechanically made on-site using machines, and two were completely hand-made, also known as “artisanal tortillas”. There were two samples of tortillas made of industrial maize flour (TMN1 and TMN2); two made of nixtamalized maize at the same place of production (TMZ1 and TMZ2); and two samples in which 50% of nixtamalized maize was substituted with industrialized maize flour (TMX1 and TMX2). For the TMZ1 tortillas, maize from the north region of Mexico was used (perhaps from Sinaloa, a region that uses technological packages for hybrid and transgenic maize massive cultivation), while the TMZ2 were made of maize from the central region of the country (Hidalgo) and a tortilla preservative was added. One of the artisanal tortillas contained a small quantity of wheat flour (TA1) and was baked on a gas cooker, while the other one (TA2) was made only of maize from the same region, Tlazala, in a traditional way using firewood. The samples were kept in paper and polyethylene bags at 4 °C until the experiments were performed. The samples were dried at 55 °C for 24 h and grounded in a Tomas Willy mill with a 0.84 mm sieve. The dried sample flours were kept in glass containers until analysis were performed.

### 2.3. Sensory and Subjective Textural Parameters

The sensory profile of fresh tortillas samples was performed following an adaptation of the lexicon and parameters assessed by Bejosano et al. [22], with the participation of nine semi-trained gastronomy specialists with prior training, with at least 3 replications, in 3 sessions of approximately 40 min, using a 15 point scale to value the intensity of each attribute. Parameters evaluated included color uniformity, surface uniformity, moisture, opacity, maize smell, lime smell, fermented smell, acid taste, salted taste, sweet taste, lime taste, roughness, elasticity, hardness, masticability, moisture absorption, and tooth packing (Table 2). The subjective textural parameters were evaluated according to the method described by Meilgaard, Vance Civille, and Carr [24] with some modifications. The descriptive spectrum method with references points was used, the scale and the anchors being adapted for the tortilla product characterization. Panelists were provided with one coded sample of tortilla once, on the same day the tortillas were made. A randomized block design was used to arrange the serving order of tortillas and the samples were distributed into individual plates. Drinking water and apple pieces were used to cleanse the mouth between samples. 

Roughness is defined as the amount of irregularities, protrusions, grains, or bumps which appear on the surface of the product [24]. Elasticity shows the degree to which the sample returns to its original shape at partial compression without breaking [22]. Hardness is the force applied to achieve a given deformation [22,24]. The moisture absorption refers to the amount of saliva absorbed by the sample during the chew down [24]. Tooth packing is a measure of the degree to which the sample sticks on the surface of teeth [24]. The rollability test was performed by rolling the sample around a 13 mm diameter dowel and the breakings were evaluated using a 15 points scale [22].

### 2.4. Chemical, Physical, and Objective Textural Parameters

The proximate composition of the tortilla samples was achieved according to the USA Association of Official Analytical Chemists methods [25]. Moisture content was assessed by sample drying at 105 °C for 24 h (method 925.098), ash content by incineration at 550 °C (method 923.03), fat content by Soxhlet extraction in petroleum ether (method 920.39C), and proteins content (N × 6.25) by the Kjeldahl method. Neutral detergent fibers (FNDs) and acid detergent fibers (FADs) were determined according to the filter bag method using an ANKOM 200 fibers analyzer [26]. Total carbohydrate content was calculated by difference. Water absorption and soluble solids indexes were evaluated according to the method described by Serena-Saldivar [27]. All measurements were performed in triplicate. 

The color profile (L*, a*, and b*) of fresh tortilla samples at room temperature was measured using a Konica Minolta Chroma Meter CR-410 and the hue angle (H*) and chroma (C*) were calculated [28]. Three measurements points for three fresh tortillas samples of each type were performed. 

Tortillas tensile strength at break and breaking distance was estimated according to the method described by Vaca-Garcia et al. [9] using a TA-TX Plus Texture Analyser (Stable Micro Systems, Godalming, United Kingdom). Circular sample pieces of 6.5 cm diameter were held by two metallic plates with a circular perforation of 2.54 cm diameter, and a spherical probe of 0.635 cm (PO25S) diameter moved through the circular perforation at a rate of 10 mm s^−1^ until the sample broke. The breaking distance and the tensile strength at break were recorded. Nine measurements for each fresh sample were performed.

### 2.5. Statistical Analysis

The statistical software SPSS version 13.0.0 for Windows (SPSS, Chicago, IL, USA) was used for the treatment of data and statistical tests. Results were reported as mean value ± standard deviation. Analysis of variance (ANOVA) was performed to determine differences between means by using Tukey’s test at a 95% confidence level. Statistically significant differences were considered at *p* < 0.05. Correlation coefficients (*r*) were determined by Pearson correlation matrix method. Principal component analysis (PCA) was carried out in order to evaluate the relationships among the studied variables and to visualize the similarities between these. Non-parametric Mann–Whitney tests were applied in order to identify the statistical differences (*p* < 0.05) among women and men in relation to the variables referring to tortilla preferences, purchase, consumption quantity, family size, purchase reasons, and purchase decision factors.

## 3. Results

### 3.1. Consumers Preferences

In this study, all participants who completed the questionnaire were tortilla consumers, the mean age being 37 years. The first part of the questionnaire contained information about preferences and purchase, while the second part was about factors influencing the purchase decision. Regarding maize type, 90% of women and men preferred tortillas made of nixtamalized maize in the purchase place, while only 63.33% of women and 73.33% of men buy it near the market which offers mostly machine tortillas made of industrialized maize flour or mixes of maize and industrialized flour. More than 90% of women and men reported consuming tortillas on a daily basis, mostly at lunch, with the majority of women consuming less than 5, while the men consumed more than 6 per day. More than 70% of the women and men buy tortillas every day, the quantity depending on the family size. Eighty percent of women and 90% of men buy tortillas from tortillerías, mostly the white variety, women because they are more common and men because they like them more. However, women reported to prefer blue tortillas, while men preferred the white ones. Regarding the factors that influence the purchase decision, the women thought that the most important was maize type, followed by maize origin, manufacturing, taste, shelf-life, appearance, and, lastly, price. Conversely, men considered taste to be the most important factor, followed by maize type, maize origin, manufacturing, price, appearance, and finally shelf-life. 

To highlight the relationship among the 12 components explored in relation to the consumers’ preferences and purchase decision, a multivariate analysis was performed using PCA as an extraction method [29]. For the first part of the questionnaire, regarding preferences and purchase reasons, eight of the components were omitted from the results of the commonalities. Four components (Table 3) contributed the most and explained 71.44% of the total variance of the model. The first component (reason for color tortilla purchase) explained 29.66% of the total variance, the second one (tortilla type purchase) explained 17.48%, the third one (family size) 13.12%, and the last one (purchase place) explained 11.17%. For the second part, even if seven components were generated, only the first four satisfied the selection criteria (eigenvalue = 1), contributing mostly (80.63%) to the explained variance of the analyzed data (Table 3). The first component (maize origin) explained 27.921% of the total variance, the second one (maize type) 20.90%, the third one (tortilla manufacture type) 16.719%, and the last one (appearance) 15.09%.

To see if there were differences between the female and male group preferences and purchase decisions, the Mann–Whitney test was applied. According to the results presented in Table 4, the favorite tortillas and the tortillas that women purchase did not differ significantly (*p* > 0.05) from those ones of the men. Also, the consumption frequency did not differ significantly (*p* > 0.05) among groups. There were significant differences (*p* < 0.01) at the medium effect size (*r* = 0.37) regarding the consumption quantity, U = 263.50, z = −2.88. The time of highest consumption differed among groups, U = 377.00, z = 2.25, *p* < 0.05. The effect size (*r* = 0.29) corresponded to a low to medium effect of the gender variable on the time of highest consumption, according to the Cohen [30] criteria. The purchase frequency and quantity, family size, purchase place, favorite color of tortillas, the actual color of purchased tortillas, and the reason for color purchase did not differ significantly (*p* > 0.05) between the female and male groups. Small-to-medium effects sizes were recorded for all factors.

According to the results presented in Table 4, there were no significant differences (*p* > 0.05) between the factors that influences women’s and men’s purchase decisions. There were weak effect sizes in all cases (*r* < 0.30).

### 3.2. Sensory, Texture Profile, and Physico-Chemical Parameters of Tortillas 

The sensory parameters of the tortilla samples are presented in Table 5. Artisanal tortillas did not have as much of a uniform color and surface as the industrial ones due to the manufacturing process [31]. Higher moisture perception and opacity were recorded for the artisanal samples. 

The smell of maize was more present in the artisanal (TA1 and TA2) and maize tortillas (TMZ1 and TMZ2), while the smell of lime was more intense for the tortillas containing industrialized maize flour. The taste of ferment and acid was not so present in any sample. The salted taste was due to the presence of natural salts in maize grains because no salt is added in the manufacturing process. A sweet taste was more pronounced in the artisanal tortillas, while the taste of lime was stronger in the industrialized samples. 

The texture parameters of the tortillas evaluated using the subjective methods are showed in Table 5. According to the obtained results, the artisanal tortilla TA1 was the roughest, while the maize tortilla TMZ2 was the softer. All samples had good rollability, the less rollable being the mix tortilla TMX1. The most elastic were the artisanal TA1 and the industrialized flour tortilla TMN1. The maize tortilla TMZ1 was the hardest, while the less one was made of industrialized flour (TMN2). Good masticability values were obtained for the artisanal and the industrial flour samples. No significant differences (*p* > 0.05) were recorded between samples regarding the moisture absorption and tooth packing parameters. 

The color of food products influences consumers preferences and can be expressed as lightness (L*), hue angle (H*), and color saturation index (chroma) [27,32,33]. According to the results presented in Table 6, the values of lightness of the artisanal tortillas samples were lower than those of the industrialized ones. Significant differences (*p* < 0.05) in the hue angle were recorded between the artisanal and the machine-made tortillas, the highest values being for TA1 and TA2. Great saturation indexes (chroma) were recorded for the samples containing industrialized maize flour, while for the maize tortillas the values were lower. 

The texture profile parameters of the tortillas achieved using the instrumental method (Table 5) depended on the maize type and manufacturing process. Higher tensile strength at break values was obtained for artisanal samples, while the mix sample TMX1 had the lower value. The breaking distance was significantly higher (*p* < 0.05) for the artisanal tortillas than for the industrial ones. 

The chemical parameters of the tortilla samples are presented in Table 7. The artisanal tortillas had the higher proteins contents compared to the industrial ones. The lipids contents varied in function of the maize origin and manufacturing process, the higher value being recorded for TMX1 sample made of industrial flour and nixtamalized maize, while the maize tortilla TMZ2 had the lower lipid content. Artisanal tortillas samples proved to have higher carbohydrates content, while the industrialized ones made with machines (TMN) had the lower content. 

The neutral detergent fibers (FND) included cellulose, hemicelluloses, and lignin [34] and had the greatest values for the mixed tortilla TMX1 (made of maize grains and industrialized corn flour) and the artisanal one which contained maize and wheat (TA1). The acid detergent fiber (FAD) included cellulose and lignin [33] and presented higher values for the artisanal tortillas than for the industrial ones (Table 7). Samples containing industrialized corn flour (TMX and TMN) presented higher values of ash contents than the other ones. Artisanal tortillas had a lower water content than samples made in tortillerías shops. The artisanal tortilla TA1 had the higher water absorption index (WAI) value, while TA2 had the lowest water absorption index. Lower water soluble solids index (WSI) values were recorded for the tortilla made of local maize only (TMZ1), followed by the industrialized ones (TMN1 and TMN2), while the artisanal (TA2) had a high-water soluble solids index. 

### 3.3. Multivariate Analysis

**Figure 1:**
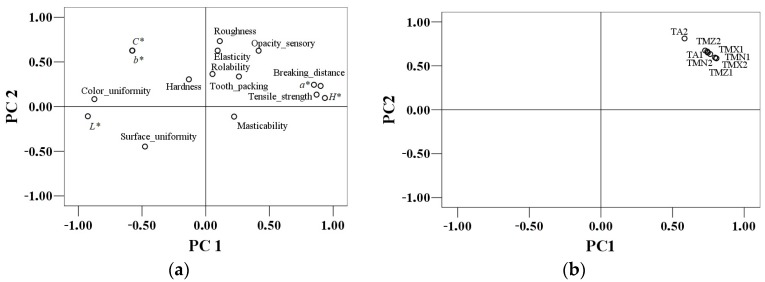
Principal component analysis: distribution of the physical (L*, a*, b*, C*, H*-color parameters, tensile strength, breaking distance), textural (hardness, roughness, elasticity, masticability, tooth packing, rollability), and sensory parameters (opacity, surface uniformity, color uniformity) analyzed (**a**). Distribution of tortillas samples (TMX—tortillas from maize grains and industrialized corn flour; TMZ—tortillas from locally grinded and nixtamalized maize grains only; TMN—tortillas from industrialized maize flour; and TA—artisanal hand-made tortillas) in the function of the analyzed parameters (**b**).

The multivariate analysis showed many effects compared to ANOVA because it takes into consideration the correlations among the dependent variables [35]. The relationships between physical (L*, a*, b*, C*, H*-color parameters, tensile strength, breaking distance) and textural parameters (hardness, roughness, elasticity, masticability, tooth packing, rollability) and the results of the sensory testing of the tortilla samples analyzed (opacity, surface uniformity and color uniformity) are shown in Figure 1a. The first two principal components explain 55.17% of the total variance (PC1 = 38.18% and PC2 = 16.98%). For the first principal component PC1, a good correlation was obtained between luminosity and color uniformity evaluated by sensory analysis (*r* = 0.78), between hue angle and a* parameter (*r* = 0.82) and between tensile strength and breaking distance (*r* = 0.87), the correlation coefficients being significant at 0.01 level. The first principal component PC1 was associated with tensile strength, breaking distance, color parameters, a*, and hue angle. The PC1 underlines the opposition between color uniformity and hue angle. As for the second principal component PC2, b*, and C* parameters were opposed to opacity characteristic. The PCA loadings showed, along PC2, a close association between roughness and elasticity, which reflects the highly significant correlation coefficient (*r* = 0.44, *p* < 0.01). These variables indicate a strong correlation with this component that can be described as a function of the sensory parameters which have an important role in assessment of tortilla samples. The second component PC2 distinguished the roughness and surface uniformity which were opposed. Significant indirect correlations were obtained between roughness and surface uniformity (*r* = −0.46, *p* < 0.01). Regarding PC2, the roughness, elasticity, opacity, tooth packing, braking distance, tensile strength, and hue angle were placed in the left of the graph which shows that these contribute to a larger extent to the evaluation of tortilla samples in comparison with the parameters on the right.

The distribution of all tortilla samples in the function of the physical, textural, and sensory analyzed parameters is presented in Figure 1b. The first two principal components explain 98.87% of the total variance (PC1 = 97.93% and PC2 = 0.93%). There were strong significant correlations at 0.01 levels among all the tortilla samples evaluated. Clustering of the samples TMZ1, TMZ2, TMX1, TMX2, TMN1, TMN2, and TA1 can be interpreted as an indication of similarity between the interrelated physical, textural, and sensory parameters. Based on the bi-plot of the principal component scores, the artisanal TA2 sample appeared quite distinct from all the other samples (Figure 1b), suggesting its differences among the evaluated parameters. 

**Figure 2:**
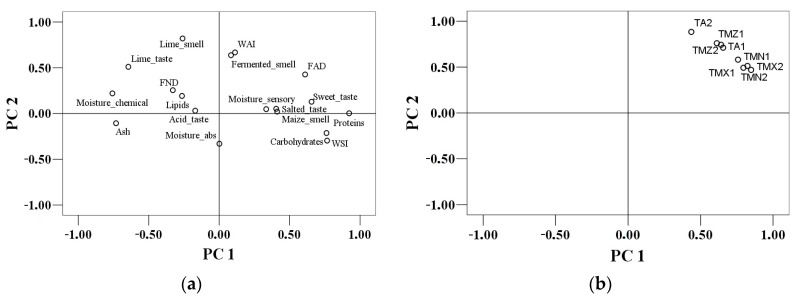
Principal component analysis: distribution of chemical (moisture, ash, proteins, lipids, neutral detergent fiber (FND), acid detergent fiber (FAD), carbohydrates, water absorption index (WAI), water soluble solids index (WSI)) and sensory (moisture absorption, acid taste, lime taste, lime smell, fermented smell, moisture sensory, salted taste, sweet taste, maize smell) analyzed parameters (**a**). Distribution of tortillas samples (TMX—tortillas from maize grains and industrialized corn flour; TMZ—tortillas from locally grinded and nixtamalized maize grains only; TMN—tortillas from industrialized maize flour; and TA—artisanal hand-made tortillas) in the function of the analyzed parameters (**b**).

In Figure 2a, the PCA loadings of the chemical (moisture, ash, proteins, lipids, neutral detergent fiber (FND), acid detergent fiber (FAD), carbohydrates, water absorption index (WAI), water soluble solids index (WSI)) and sensory parameters (moisture absorption, acid taste, lime taste, lime smell, fermented smell, moisture sensory, salted taste, sweet taste, maize smell) are presented. A value of 41.53% of the total variance was explained by the first two principal components (PC1 = 29.04% and PC2 = 12.48%). There were statistically significant correlations (*p* < 0.01) between neutral detergent fibers (FNDs) and lipids (*r* = 0.55), maize smell and salted taste (*r* = 0.45) and water absorption index (WAI) (*r* = 0.34), and carbohydrates and water soluble solids index (WSI) (*r* = 0.57). The first principal component was associated with protein content, sweet taste, WSI, and carbohydrates, while the second one was associated with fermented smell, WAI, and lime smell. Along the PC1 axis, the ash content and moisture measured chemically was opposed to proteins content, WSI, and carbohydrates. The PC2 distinguished between moisture absorption and WAI with the latter showing a positive effect on the fermented smell. From the PCA bi-plot in Figure 2a, the correlations between the results of the chemical and sensory evaluation for the tortillas samples are observed.

The scores were also denoted for each tortilla sample and grouped corresponding to their manufacturing process type (Figure 2b). The first two principal components explain 93.66% of the total variance (PC1 = 90.04% and PC2 = 3.62%). Samples grouping (Figure 2b) shows that, probably, the processing method did not remarkably influence the tortilla quality considered from the point of view of the sensory and chemical parameters. The TA1 sample was visibly differentiated from the others, a fact that may be attributed to the use of an amount of wheat flour in the formulation recipe, the ingredients used being a critical factor that affects tortilla quality [36]. Apart of TA1, two more sample groups can be distinguished. The first one included the samples made of only of maize grains (TMZ1, TMZ2m and TA1), while the second one comprised the samples with industrial flour (TMX1, TMX2, TMN1, and TMN2). Regarding PC2, the WAI, FAD, fermented smell, sweet taste, salted taste, maize smell and the moisture sensory evaluated are placed on the left of the graph which shows that these contributed to a larger extent at the evaluation of tortillas samples in comparison with the parameters on the right. There were strong correlations among all tortillas samples made with industrialized corn flour, either alone (TMN) or mixed with local maize flour (TMX) (Figure 2b), between TMX2 and TMX1 (*r* = 0.86), TMX2 and TMN2 (*r* = 0.87), and TMX1 and TMN2 (*r* = 0.85) at the 0.01 significance level. However, artisanal TA1 was strongly correlated with tortillas made from locally grinded and nixtamalized maize grains TMZ2 (*r* = 0.93) and TMZ1 (*r* = 0.93) at the 0.01 significance level and was characterized by high WAI. 

## 4. Discussion

The hypothesis that there are differences between women’s and men’s preferences and purchase decisions was confirmed by the results obtained for the factors affecting the purchase decision; men considered taste as the most important fact while, for women the maize type was essential. Another study mentioned that food choice is a complex process which depends on gender influences [37]. Significant differences (*p* < 0.01) among the two groups were obtained for the consumption quantity and time of highest consumption of tortillas which is due to the distinct energy needs between women and men [19]. Women are reported to be the main decision-makers for food purchase [38] and household economics, trying to offer pragmatic responses when spending on food for their families, as it is for the purchase of tortillas. Several studies revealed that the nutritional value of the product is an important factor that affects consumer’s behavior [38,39,40,41,42]. Our results showed that women consume less than 5 pieces of tortillas daily, while men consume more than 6, and this is in accordance with the findings reported by Cárdenas-Marcelo et al. [15] which stated that women consume approximately seven tortillas daily and men nine. Our study revealed that 80% of women and 90% of men buy tortillas from tortillerías, while Cárdenas-Marcelo et al. [15] reported that only 16% of women and 9% of men buy them from such places. These differences may be due to the fact that our study was performed in the center of the village of Tlazala which is close to the urban area of Mexico City, while their study was conducted in a local market where artisanal products are sold, in the State of Mexico. The most important factors that influence consumers from Tlazala tortillas selection were the maize type, origin, manufacture, and taste. The reasons for their choices were related to the preference or to the product availability which implies the proximity of their houses and the variety of products, a fact also confirmed by other studies [38,43,44]. 

Characteristics such as taste, smell, appearance, and texture of food products have a determinant role when a person decides to buy and consume an item [45]. The obtained results showed higher scores for the sensory parameters such as moisture, opacity, maize smell, and sweet taste, and lower scores for lime taste and the smell of the artisanal and maize made tortillas which are in agreement with the results for the consumers’ preferences. Similar findings were reported by Méndez-Albores et al. [31] for maize tortillas with different nixtamalization processes when panelists detected the lime smell and taste at 0.50% (*w*/*w*) lime used. According to the texture parameters scores, the artisanal tortilla TA1 roughness was appreciated with 5.83 points and the elasticity with 7.89, being higher compared to most of the industrial ones studied. All the samples presented high scores for rollability and masticability, while the hardness varied from 3.66 for TMN2 made of industrial flour to 7.33 for the TMZ2 industrially made of maize grains. These results are near to those obtained by Bejosano et al. [22] for wheat flour tortillas which had a rollability score of 15.00, elasticity of 11.10, and hardness of 6.50, measured using a 15 points scale. Méndez-Albores et al. [31] reported roughness scores between 4.77 and 4.90, rollability between 4.19 and 4.51, tooth packing between 4.87 and 4.90 for maize tortillas evaluated using a 10 point scale. The relationship between the sensory profile of food products and consumers’ behavior has been previously described in the literature [46,47,48]. 

Higher nutritional value and satisfactory texture parameters obtained for the tortillas made of maize grains instead of industrial flour sustain the preferences of consumers for this kind of product. Genetic and environmental conditions affect both the chemical and physical parameters of maize grains and, thus, the tortillas quality [42]. The textural properties are determined by the competition of starch, proteins, and fibers for water, influencing the starch matrix formation and starch grain hydration and plasticization. Thus, the chemical composition of tortillas is responsible for the textural property changes [10]. The chemical and physical parameters of tortillas depend on the maize variety, nixtamalization, and manufacturing process. Our results showed a lower lightness value for the artisanal tortillas, in agreement with the findings reported by Khan et al. [32] for traditional and machine tortillas. The hue angle of the artisanal tortillas was higher than 266, while the industrial ones varied from 91.92 to 95.19. Our results showed higher values than those by Vasquez-Carrillo et al. [33] for industrial tortillas which presented values between 70.00 and 82.90, probably due to the maize type and manufacturing process. The tensile strength at the highest break value (1064.75 N) for the artisanal tortilla TA1 compared to the industrial ones was in agreement with that obtained by Vaca-García et al. [9] which showed a value of 1684.00 N for the maize tortilla. The artisanal tortillas had the highest protein content of 9.33 g/100 g for TA1 and 9.52 g/100 g for TA2, the results being in agreement with those obtained by Valderrama-Bravo et al. [10] for tortillas from different maize genotypes which presented values higher than 11 g/100 g. The lipid contents of the studied tortillas were lower than those reported by Valderrama-Bravo et al. [10] and can be due to the fat hydrolysis in alkaline solution and solubilization during the nixtamalization process [33]. The artisanal tortilla TA2 had the lowest water absorption index. As water content influences texture, lower values lead to harder and brittle tortillas which cannot support a stew portion on top of it, which is the most common way to consume tortillas in Mexico [33]. The artisanal tortillas presented the highest fiber and lower ash contents which are in agreement with Valderrama-Bravo et al.’s [10] previous study. Therefore, our results highlighted that the chemical composition of the artisanal tortillas recommend them as being more nutritious compared to the industrial ones. 

This study underlines the properties of some tortilla types and people’s preferences from a specific region in Mexico. However, it may represent a starting point for future investigations regarding the relationships between tortilla quality and consumer behavior.

## 5. Conclusions

The industrialization of the food sector leads to product changes. Tortillas parameters and consumers’ behavior knowledge is important for all implied actors, from consumer to producer. Consumers’ preferences for artisanal maize tortillas instead of industrialized maize flour were supported by the quality parameters of these products. The artisanal tortillas stand out from the industrial ones by higher nutritional value and proper sensory parameters. Tortillas made of maize processed in a mechanized way had satisfactory physical, chemical, and sensory parameters compared to those made of industrialized flour and, thus, can be considered as an intermediate choice between the artisanal and industrialized ones. In Tlazala, no significant differences were found between women’s and men’s behavior regarding tortilla choice, except for purchase decision factors. Women agreed that the most important aspect was the maize type, while men were more interested in the product’s taste. People search for healthy and convenient products with good parameters, and producers should be focused on product quality improvement and consumer need satisfaction. 

## Figures and Tables

**Table 1:** Questionnaire items that consumers could choose.

Section	Dimension	Items
Purchase and preferences	Tortilla type preference	I prefer tortillas made of maize.
		I prefer tortillas made of industrial flour.
		I prefer tortillas made of mix of maize and industrial flour.
	Tortillas type purchase	I buy tortillas made of maize.
		I buy tortillas made of industrial flour.
		I buy tortillas made of mix of maize and industrial flour.
	Consumption frequency	I consume tortillas daily.
		I consume tortillas every 2–3 days.
		I consume tortillas weekly.
		I consume tortillas monthly.
		I never consume tortillas.
	Consumption quantity	I consume 1 or 2 tortillas a day.
		I consume between 3 and 5 tortillas a day.
		I consume between 6 and 10 tortillas a day.
		I consume more than 10 tortillas a day.
	Time of highest consumption	I consume tortillas the most at breakfast.
		I consume tortillas the most at lunch.
		I consume tortillas the most at dinner.
		I consume tortillas the most at snack.
	Purchase frequency	I buy tortillas daily.
		I buy tortillas every 2–3 days.
		I buy tortillas weekly.
		I buy tortillas monthly.
		I never buy tortillas.
	Purchase quantity	0.5 to 3 kg (open question)
	Family size	My family size is of 1 or 2 persons.
		My family size is of 3 or 4 persons.
		My family size is of 5 or 6 persons.
		My family size is of more than 7 persons.
	Purchase place	I usually buy tortillas from supermarket.
		I usually buy tortillas from shop.
		I usually buy tortillas from tortillerías.
		I usually buy tortillas from particular houses.
		I make tortillas home.
	Color preference	I prefer white tortillas.
		I prefer yellow tortillas.
		I prefer blue tortillas.
		It does not matter.
	Tortilla color type purchase	I buy white tortillas.
		I buy yellow tortillas.
		I buy blue tortillas.
		It does not matter.
	Reason for color tortillas purchase	They are the most popular.
		I like them.
		It is nearby my house.
Factors influencing the purchase decision	Maize origin	
	Maize type	
	Tortillas manufacture type	
	Tortillas appearance	
	Price	
	Taste	
	Shelf-life	

**Table 2:** Sensory analysis and subjective textural characteristics scores [22,24].

Name	Score Sheet for Descriptive Analysis of Tortilla Samples
Color uniformity	1 = nothing uniform, 15 = very uniform
Surface uniformity	1 = nothing uniform, 15 = very uniform
Moisture	1 = nothing moist, 15 = very moist
Opacity	1 = translucent, 7 = a little opaque, 15 = very opaque
Maize smell	1 = nothing intense, 15 = very intense
Lime smell	1 = nothing intense, 15 = very intense
Fermented smell	1 = nothing intense, 15 = very intense
Acid taste	1 = nothing acid, 15 = very acid (2 = mineral water, 7.5 = orange juice, 15 = lime)
Salted taste	1 = nothing salted, 15 = very salted (8 = crackers, 13 = potato chips)
Sweet taste	1 = nothing sweet, 15 = very sweet (6 = orange juice, 9 = cola juice)
Lime taste	1 = nothing intense, 15 = very intense
Roughness	1 = nothing rough, 15 = very rough (1 = gelatin, 5 = orange peel, 8 = potato chips, 12 = hard granola bar)
Rollability	1 = nothing rollable, 15 = it rolls without breaking (1 = breaks along the axis, 7 = it breaks on both sides, 11 = it breaks on one side)
Elasticity	1 = nothing elastic, 15 = very elastic (1 = cheese cream, 5 = sausage, 9.5 = marshmallow, 15 = gelatin)
Hardness	1 = soft, 15 = hard (1 = cheese cream, 4.5 = cheese, 6 = olive, 9 = peanuts, 11 = almonds)
Masticability	1 = nothing chewable, 15 = very chewable
Moisture absorption	1 = it does not absorb water, 15 = it absorbs a large quantity of water (1 = candy, 7.5 = popcorn, 10 = potato chips, 15 = biscuits)
Tooth packing	1 = nothing sticky, 2 = very sticky (1 = carrot, 9 = cheese, 15 = gummy candy)

**Table 3:** PCA with the correspondent eigenvalues and the percentages of variance explained.

Component	Name	Eigenvalues	Total Explained Variance	
			% of Variance	% Accumulated
Purchase and preferences				
Component 1	Reason for color tortilla purchase	2.67	29.66	29.66
Component 2	Tortillas type purchase	1.57	17.48	47.15
Component 3	Family size	1.18	13.12	60.27
Component 4	Purchase place	1.00	11.17	71.44
Factors influencing purchase decision				
Component 1	Maize origin	1.95	27.92	27.92
Component 2	Maize type	1.46	20.90	48.82
Component 3	Tortillas manufacture type	1.17	16.71	65.54
Component 4	Tortillas appearance	1.05	15.09	80.63

**Table 4:** Mann–Whitney test results for the female and male group comparison regarding tortilla preferences and purchase decision.

Factor	Median	Range	Minimum	Maximum	U	z	*r*	Significance
Purchase and preferences								
Favorite tortillas	1.00	2.00	1.00	3.00	448.50	−0.04	0.01	0.96
Purchased tortillas	1.00	2.00	1.00	3.00	411.50	−0.69	0.09	0.48
Consumption frequency	1.00	1.00	1.00	2.00	435.00	−0.58	0.07	0.55
Consumption quantity	3.00	3.00	1.00	4.00	263.50	−2.88	0.37	0.00
Time of highest consumption	2.00	2.00	1.00	3.00	377.00	−2.25	0.29	0.02
Purchase frequency	1.00	4.00	1.00	5.00	439.00	−0.20	0.02	0.83
Purchase quantity	3.00	6.00	1.00	7.00	411.50	−0.61	0.08	0.53
Family size	2.00	3.00	1.00	4.00	434.00	−0.25	0.03	0.80
Purchase place	3.00	4.00	1.00	5.00	377.00	−1.741	0.22	0.08
Color of favorite tortilla	3.00	6.00	1.00	7.00	355.50	−1.43	0.18	0.15
Color of tortilla purchase	2.00	6.00	1.00	7.00	417.50	−0.51	0.06	0.60
Reason of color tortilla purchase	2.00	3.00	1.00	4.00	422.50	−0.43	0.05	0.66
Factors influencing purchase decision								
Price	6.00	6.00	1.00	7.00	367.00	−1.29	0.16	0.19
Taste	3.00	6.00	1.00	7.00	331.00	−1.80	0.23	0.07
Tortillas manufacture	4.00	6.00	1.00	7.00	401.50	−0.72	0.09	0.46
Tortillas appearance	5.00	6.00	1.00	7.00	435.00	−0.22	0.02	0.82
Shelf-life	5.00	6.00	1.00	7.00	352.00	−1.47	0.19	0.13
Maize type	3.00	6.00	1.00	7.00	397.00	−0.79	0.10	0.42
Maize origin	3.00	6.00	1.00	7.00	420.00	−0.45	0.05	0.65

U: Mann–Whitney; z: z score; *r*: the effect size; median: measure of the central tendency of the two groups; range: median dispersion.

**Table 5:** Sensory and subjective textural parameters of tortilla samples.

Sensory Characteristic	Sample							
	TMX1	TMX2	TMZ1	TMZ2	TMN1	TMN2	TA1	TA2
Color uniformity	12.00 ± 1.39 ^bc^	10.77 ± 1.38 ^bc^	10.77 ± 1.71 ^bc^	9.66 ± 2.54 ^b^	10.88 ± 1.52 ^bc^	12.22 ± 0.57 ^c^	6.11 ± 1.63 ^a^	5.66 ± 2.87 ^a^
Surface uniformity	9.44 ± 2.22 ^abc^	8.00 ±2.23 ^ab^	10.55 ± 1.77 ^bc^	11.77 ± 1.82 ^c^	11.77 ± 1.67 ^c^	9.44 ± 2.22 ^abc^	6.55 ± 2.87 ^a^	7.55 ± 2.85 ^ab^
Moisture	7.33 ± 2.57 ^a^	6.33 ±1.98 ^a^	6.22 ± 2.73 ^a^	8.11 ± 2.26 ^a^	8.00 ± 2.16 ^a^	8.22 ± 1.49 ^a^	9.33 ± 3.27 ^a^	8.11 ± 2.30 ^a^
Opacity	7.66 ± 1.25 ^ab^	9.11 ± 2.08 ^bc^	8.88 ± 2.19 ^bc^	6.00 ± 2.36 ^a^	7.77 ± 2.57 ^ab^	9.22 ± 1.70 ^bc^	11.88 ± 1.34 ^c^	9.77 ± 1.57 ^bc^
Maize smell	6.33 ± 2.69 ^ab^	4.88 ± 3.49 ^a^	7.33 ± 3.14 ^ab^	8.55 ± 2.58 ^ab^	7.66 ± 2.14 ^ab^	5.77 ± 3.03 ^ab^	10.00 ± 2.50 ^b^	8.88 ± 3.38 ^ab^
Lime smell	8.00 ± 3.67 ^bc^	8.66 ± 3.55 ^c^	5.77 ± 4.03 ^abc^	3.55 ± 2.14 ^ab^	9.89 ± 2.81 ^c^	8.89 ± 3.26 ^c^	4.33 ± 3.03 ^c^	3.55 ± 2.13 ^a^
Fermented smell	3.22 ± 1.11 ^a^	3.00 ± 2.57 ^a^	4.22 ± 1.67 ^a^	3.66 ± 1.25 ^a^	4.00 ± 2.93 ^a^	4.55 ± 2.60 ^a^	3.44 ± 1.27 ^a^	2.11 ± 1.21 ^a^
Acid taste	2.55 ± 1.13 ^a^	4.00 ± 2.07 ^a^	2.66 ± 1.61 ^a^	2.89 ± 1.70 ^a^	1.89 ± 0.78 ^a^	3.66 ± 2.69 ^a^	2.66 ± 1.86 ^a^	2.33 ± 1.46 ^a^
Salted taste	2.66 ± 0.97 ^a^	3.44 ± 2.11 ^a^	3.33 ± 1.82 ^a^	3.66 ± 1.13 ^a^	2.44 ± 0.97 ^a^	3.22 ± 0.81 ^a^	3.55 ± 1.27 ^a^	2.78 ± 1.13 ^a^
Sweet taste	4.00 ± 1.57 ^abc^	1.67 ± 0.53 ^a^	3.44 ± 0.81 ^ab^	3.78 ± 1.27 ^abc^	3.89 ± 1.39 ^abc^	2.44 ± 1.13 ^a^	5.44 ± 2.26 ^bc^	5.44 ± 1.27 ^c^
Lime taste	5.89 ± 3.28 ^ab^	11.11 ± 2.87 ^c^	5.11 ± 2.22 ^ab^	4.55 ± 2.03 ^ab^	8.11 ± 4.02 ^bc^	8.78 ± 2.92 ^bc^	3.78 ± 2.38 ^a^	3.44 ± 1.95 ^a^
Roughness	5.00 ± 1.06 ^ab^	5.39 ± 1.01 ^ab^	4.94 ± 2.14 ^ab^	2.55 ± 0.37 ^a^	4.55 ± 0.48 ^ab^	5.22 ± 1.52 ^ab^	5.83 ± 3.65 ^b^	4.11 ± 1.86 ^ab^
Rollability	10.44 ± 3.82 ^a^	13.11 ± 1.70 ^a^	11.89 ± 1.70 ^a^	12.55 ± 1.27 ^a^	12.22 ± 4.48 ^a^	13.89 ± 1.57 ^a^	14.11 ± 0.78 ^a^	11.55 ± 1.60 ^a^
Elasticity	5.78 ± 1.79 ^a^	6.55 ± 1.73 ^a^	6.89 ± 1.15 ^a^	5.00 ± 1.41 ^a^	7.05 ± 2.49 ^a^	6.44 ± 1.13 ^a^	7.89 ± 3.43 ^a^	5.33 ± 2.38 ^a^
Hardness	3.89 ± 0.95 ^a^	5.89 ± 1.98 ^a^	7.33 ± 4.25 ^a^	3.78 ± 1.11 ^a^	4.05 ± 0.83 ^a^	3.66 ± 0.75 ^a^	5.33 ± 1.49 ^a^	4.11 ± 1.29 ^a^
Masticability	7.55 ± 4.11 ^a^	7.77 ± 3.59 ^a^	8.89 ± 2.21 ^a^	10.77 ± 2.21 ^a^	10.11 ± 2.07 ^a^	10.44 ± 1.70 ^a^	9.66 ± 2.70 ^a^	10.11 ± 2.92 ^a^
Moisture absorption	8.55 ± 2.76 ^a^	11.11 ± 1.57 ^a^	10.77 ± 1.77 ^a^	9.11 ± 2.60 ^a^	8.20 ± 3.26 ^a^	9.66 ± 3.31 ^a^	11.11 ± 2.81 ^a^	9.55 ± 2.00 ^a^
Tooth packing	6.00 ± 3.49 ^a^	8.22 ± 4.37 ^a^	8.78 ± 3.14 ^a^	7.44 ± 2.13 ^a^	6.89 ± 3.43 ^a^	7.33 ± 3.43 ^a^	9.44 ± 3.09 ^a^	7.89 ± 2.60 ^a^

TMX: tortillas from maize grains and industrialized corn flour; TMZ: tortillas from locally grinded and nixtamalized maize grains only; TMN: tortillas from industrialized maize flour; TA: artisanal hand-made tortillas. The results are reported as the mean of at least three replications. Means with the same letters in the same row are not significantly different (Tukey *p* < 0.05).

**Table 6:** Color and mechanical texture parameters of tortillas samples.

Sample	L*	H*	C*	Tensile Strength at Break (N)	Breaking Distance (mm)
TMX1	69.94 ± 0.81 ^de^	92.92 ± 0.85 ^ab^	19.95 ± 0.31 ^e^	438.33 ± 59.99 ^a^	6.79 ± 0.31 ^a^
TMX2	67.87 ± 0.65 ^c^	91.60 ± 0.73 ^a^	19.64 ± 0.56 ^e^	684.71 ± 86.13 ^bcd^	7.39 ± 0.27 ^a^
TMZ1	71.16 ± 0.86 ^b^	92.69 ± 1.17 ^ab^	15.30 ± 0.61 ^c^	713.26 ± 56.61 ^cd^	7.53 ± 0.39 ^d^
TMZ2	68.11 ± 0.98 ^c^	95.19 ± 1.81 ^c^	12.75 ± 0.77 ^a^	571.15 ± 70.61 ^abc^	6.61 ± 0.40 ^a^
TMN1	68.85 ± 0.68 ^cd^	91.96 ± 1.13 ^a^	20.06 ± 0.60 ^e^	551.29 ± 78.17 ^ab^	6.61 ± 0.40 ^a^
TMN2	71.07 ± 0.89 ^ef^	93.97 ± 0.63 ^bc^	19.83 ± 0.30 ^e^	457.88 ± 67.31 ^a^	6.79 ± 0.92 ^a^
TA1	58.09 ± 0.77 ^a^	266.18 ± 1.94 ^d^	16.71 ± 0.69 ^d^	1064.75 ± 71.46 ^e^	11.34 ± 0.93 ^c^
TA2	61.49 ± 0.88 ^b^	268.45 ± 0.92 ^e^	13.69 ± 0.69 ^b^	725.37 ± 45.38 ^d^	9.24 ± 0.30 ^b^

TMX: tortillas from maize grains and industrialized corn flour; TMZ: tortillas from grinded and nixtamalized maize grains only; TMN: tortillas from industrialized maize flour; TA: artisanal hand-made tortillas; L*: luminosity; H*: hue angle; C*: chroma. The results are reported as the means of nine replications. Means with the same letters in the same column are not significantly different (Tukey *p* < 0.05).

**Table 7:** Chemical parameters of tortilla samples expressed as g/100 g.

Sample	Proteins	Lipids	Carbohydrates	FND	FAD	Ash	Moisture	WAI	WSI
TMX1	7.51 ± 0.01 ^a^	1.43 ± 0.00 ^d^	37.22 ± 0.68 ^c^	33.95 ± 0.53 ^g^	0.60 ± 0.25 ^ab^	2.16 ± 0.13 ^c^	51.57 ± 0.65 ^d^	4.00 ± 0.16 ^ab^	2.80 ± 0.29 ^ab^
TMX2	7.66 ± 0.02 ^ab^	1.12 ± 0.01 ^bc^	39.57 ± 0.83 ^d^	17.31 ± 0.61 ^e^	0.76 ± 0.34 ^ab^	3.34 ± 0.04 ^e^	47.94 ± 0.87 ^bc^	3.91 ± 0.02 ^ab^	2.72 ± 0.35 ^ab^
TMZ1	7.84 ± 0.01 ^bc^	1.14 ± 0.03 ^bc^	41.06 ± 0.04 ^e^	15.44 ± 0.54 ^cd^	0.75 ± 0.09 ^ab^	1.28 ± 0.08 ^a^	48.52 ± 0.25 ^c^	4.18 ± 0.13 ^bc^	2.07 ± 0.39 ^a^
TMZ2	8.04 ± 0.01 ^cd^	0.73 ± 0.02 ^a^	38.59 ± 0.51 ^d^	16.20 ± 0.54 ^d^	0.54 ± 0.18 ^a^	1.54 ± 0.03 ^b^	50.99 ± 0.39 ^d^	4.06 ± 0.05 ^ab^	3.61 ± 0.25 ^c^
TMN1	7.65 ± 0.21 ^ab^	1.20 ± 0.16 ^c^	34.20 ± 0.66 ^b^	14.24 ± 0.51 ^b^	1.01 ± 0.15 ^ab^	2.51 ± 0.04 ^d^	54.22 ± 0.58 ^e^	3.98 ± 0.08 ^ab^	2.76 ± 0.13 ^ab^
TMN2	8.09 ± 0.09 ^d^	1.02 ± 0.05 ^b^	32.44 ± 0.82 ^a^	14.83 ± 0.26 ^bc^	0.73 ± 0.14 ^ab^	2.58 ± 0.02 ^d^	55.57 ± 0.85 ^e^	4.19 ± 0.07 ^bc^	2.36 ± 0.05 ^a^
TA1	9.33 ± 0.15 ^e^	1.13 ± 0.01 ^bc^	41.65 ± 0.48 ^e^	22.65 ± 0.37 ^f^	1.82 ± 0.18 ^c^	1.55 ± 0.08 ^b^	46.49 ± 0.28 ^b^	4.46 ± 0.01 ^c^	3.35 ± 0.27 ^bc^
TA2	9.52 ± 0.09 ^e^	1.09 ± 0.00 ^bc^	43.94 ± 0.48 ^f^	8.31 ± 0.44 ^a^	1.07 ± 0.39 ^b^	1.41 ± 0.04 ^ab^	43.71 ± 0.61 ^a^	3.80 ± 0.44 ^a^	5.14 ± 0.34 ^d^

TMX: tortillas from maize grains and industrialized corn flour; TMZ: tortillas from locally grinded and nixtamalized maize grains only; TMN: tortillas from industrialized maize flour; TA: artisanal hand-made tortillas; FND: neutral detergent fibers; FAD: acid detergent fibers; WAI: water absorption index; WSI: water soluble solids index. The results are reported as the means of at least three replications. Means showing the same letters in the same column are not significantly different (Tukey *p* < 0.05).

## References

[1] Rodríguez Calderón T.J., Chávez Mejía M.C., Thomé Ortiz H., Miranda Román G. (2017). Production and consumption of tortillas as a cultural heritage of San Pedro del Rosal, Mexico. Región Y Sociedad.

[2] Novelo V., García A. (1987). La tortilla: Alimento, trabajo y tecnología. Complementos del Seminario de Problemas Científicos y Filosóficos.

[3] Gwirtz A.J., Garcia-Casal M.N. (2014). Processing maize flour and corn meal food products. Ann. N. Y. Acad. Sci..

[4] Bourges H., Lehrer S. (2004). Assessment of Human Health Effects in Maize and Biodiversity: The Effects of Transgenic Maize in MEXICO.

[5] Pourafshar S., Rosentrater K.A., Krishnan P.G. (2015). Changes in chemical and physical properties of Latin American wheat flour based tortillas substituted with different levels of distillers dried grains with solubles (DDGS). J. Food Sci. Technol..

[6] Vizcarra Bordi I. (2018). Volteando la Tortilla.

[7] Isakson R. (2007). Between the Market and the Milpa: Market Engagements, Peasant Livelihood Strategies, and the On-Farm Conservation of Crop Genetic Diversity in the Guatemalan Highlands. Ph.D. Thesis.

[8] Sain G., Amaya N., Trejos R. (2014). Maize Situation in Latin America: Outlook and Investment Opportunities.

[9] Vaca-García V.M., Martínez-Rueda C.G., Mariezcurrena-Berasain M.D., Domínguez-López A. (2011). Functional properties of tortillas with triticale flour as a partial substitute of nixtamalized corn flour. LWT-Food Sci. Technol..

[10] Valderrama-Bravo C., Domínguez-Pacheco A., Hernández-Aguilar C., Zepeda-Bautista R., del Real-López A., Pahua-Ramos M.E., Arellano-Vázquez J.L., Moreno-Martínez E. (2017). Physical and chemical characterization of masa and tortillas from parental lines, crosses, and one hybrid. Int. Agrophys..

[11] Vizcarra Bordi I. (2002). Entre el taco Mazahua y el Mundo. La Comida de las Relaciones de Poder, Resistencia e Identidades.

[12] Appendini K., Quijada M.G. (2016). Consumption strategies in Mexican rural households: Pursuing food security with quality. Agric. Hum. Values.

[13] La percepción del consumidor de tortillas. https://www.jornada.com.mx/2018/02/17/cam-tortillas.html.

[14] Ortega T. (2018). Género, Soberanía Alimentaria y Agrobiodiversidad: La Unión de Palmeadoras de la Heroica Ciudad se Tlaxiaco, Oaxaca. Ph.D. Thesis.

[15] Cárdenas Marcelo A.L., Vizcarra Bordi I., Espinoza-Ortega A., Espinosa Calderón A. (2019). Artisanal Mazahua Tortillas and Native Maize Biodiversity. Reflexion from the Ecofeminism of Subsistence. Sociedad Y Ambiente.

[16] Torres-Salcido G. (2009). De la producción de maíz al consumo social de tortilla. Políticas de Producción y Abastecimiento Urbano.

[17] Arabi M. (2010). Linking Tortilla Price Policies to Household Food Consumption and Child Nutritional Intake: Potential Outcomes of Globalization in Rural Mexico.

[18] Herrera-Corredor J.A., Saidu J.E., Khachatryan A., Prinyawiwatkul W., Carballo-Carballo A., Zepeda-Bautista R. (2007). Identifying drivers for consumer acceptance and purchase intent of corn tortilla. J. Food Sci..

[19] Chao A.M., Loughead J., Bakizada Z.M., Hopkins C.M., Geliebter A., Gur R.C., Wadden T.A. (2017). Sex/gender differences in neural correlates of food stimuli: A systematic review of functional neuroimaging studies. Obes. Rev..

[20] Rangel Meza E., Muñoz Orozco A., Vázquez Carrillo G., Cuevas Sánchez J., Merino Castillo J., Miranda Colín S. (2004). Nixtamalización, elaboración y calidad de tortilla de maíces de ecatlán, Puebla, México. Agrociencia.

[21] Figueroa Cardenas J.D., Acero Godínez M.G., Vasco Méndez N.L., Lozano Guzman A., Flores Acosta L.M., González-Hernández J. (2001). Fortification and evaluation of nixtamal tortillas. Arch. Latinoam. Nutr..

[22] Bejosano F.P., Joseph S., Lopez M.L., Kelekci N.N., Waniska R.D. (2016). Rheological a sensory evaluation of wheat flour tortillas during storage. Cereal Chem..

[23] Méndez-Albores A., Martínez-Morquecho R.A., Moreno-Martínez E., Vázquez-Durán A. (2012). Technological properties of maize tortillas produced by microwave nixtamalization with variable alkalinity. Afr. J. Biotechnol..

[24] Morten Meilgaard D.S., Gail Vance Civille B.S., Thomas Carr M.S. (2007). Sensory Evaluation Techniques.

[25] Association of Official Analytical Chemists (AOAC) (1999). Official Methods of Analysis of the AOAC.

[26] Ankom Anal Methods. https://www.ankom.com/analytical-methods-support/fiber-analyzer-a200.

[27] Serena-Saldivar O. (2012). Cereal Grains Laboratory Reference and Procedures Manual.

[28] Hernández-Martínez V., Salinas-Moreno Y., Ramírez-Díaz J.L., Vásquez-Carrillo G., Domínguez-López A., Ramírez-Romero A.G. (2016). Color, Phenolic composition and antioxidant activity of blue tortillas from Mexican maize races. CYTA J. Food.

[29] Espinoza-Ortega A., Martínez-García C.G., Thomé-Ortiz H., Vizcarra-Bordi I. (2016). Motives for food choice of consumers in Central México. Br. Food J..

[30] Cohen J. (1988). Statistical Power Analysis for the Behavioral Sciences.

[31] Méndez-Albores A., Zamora-Rodríguez D., Arámbula-Villa G., Vásquez-Durán A., Moreno-Martínez E. (2012). Impact of different alkaline-heating processes on technological and nutritional properties of maize tortillas. J. Food Nutr. Res..

[32] Khan M.N., Des Rosiers M.C., Rooney L.W., Morgan R.G., Sweat V.E. (1982). Corn tortillas: Evaluation of corn cooking procedures. Cereal Chem..

[33] Vázquez-Carrillo G., García-Lara S., Salinas-Moreno Y., Bergvinson D.J., Palacios-Rojas N. (2017). Grain and Tortilla Quality in Landraces and Improved Maize Grown in the Highlands of Mexico. Plant Foods Hum. Nutr..

[34] Van Soest P.J., Robertson J.B., Lewis B.A. (1991). Carbohydrate methodology, metabolism and nutritional implications in dairy cattle. J. Dairy Sci..

[35] Field A. (2013). Discovering Statistics Using IBM SPSS Statistics.

[36] Rooney L.W., Serena-Saldivar S.O. (2015). Tortillas Wheat Flour and Corn Products.

[37] Woodhall-Melnik J., Matheson F. (2016). More than convenience: The role of habitus in understanding the food choices of fast food workers. Work Employ. Soc..

[38] Escobar-López S.Y., Espinoza-Ortega A., Vizcarra-Bordi I., Thomé-Ortiz H. (2017). The consumer of food products in organic markets of central Mexico. Br. Food J..

[39] Lee H.J., Yun Z.S. (2015). Consumers′ perceptions of organic food attributes and cognitive and affective attitudes as determinants of their purchase intentions toward organic food. Food Qual. Prefer..

[40] Aschemann-Witzel J., Maroscheck N., Hamm U. (2013). Are organic consumers preferring or avoiding foods with nutrition and health claims?. Food Qual. Prefer..

[41] Pérez-Villarreal H.H., Martínez-Ruiz M.P., Izquierdo-Yusta A. (2019). Testing Model of Purchase Intention for Fast Food in Mexico: How do Consumers React to Food Values, Positive Anticipated Emotions, Attitude toward the Brand, and Attitude toward Eating Hamburgers?. Foods.

[42] Vázquez-Carrillo M.G., Santiago-Ramos D., Salinas-Moreno Y., López-Cruz J., Ybarra-Moncada M.C., Ortega-Corona A. (2011). Oil content in maize (*Zea mays* L.) genotypes and its relationship with quality and texture of tortilla. Agrociencia.

[43] Lim W.M., Yong J.L.S., Suryadi K. (2014). Consumers′ perceived value and willingness to purchase organic food. J. Glob. Mark..

[44] Hjelmar U. (2011). Consumers′ purchase of organic food products. A matter of convenience and reflexive practices. Appetite.

[45] Pollard J., Kirk S.L., Cade J.E. (2002). Factors affecting food choice in relation to fruit and vegetable intake: A review. Nutr. Res. Rev..

[46] Steptoe A., Pollard T.M., Wardle J. (1995). Development of a measure of the motives underlying the selection of food: The food choice questionnaire. Appetite.

[47] Eertmans A., Victoir A., Vansant G., Van den Bergh O. (2005). Food-related personality traits, food choice motives and food intake: Mediator and moderator relationships. Food Qual. Prefer..

[48] Baudry J., Péneau S., Allès B., Touvier M., Hercberg S., Galan P., Amiot M.J., Lairon D., Mejean C., Kesse-Guyot E. (2017). Food choice motives when purchasing in organic and conventional consumer clusters: Focus on sustainable concerns (The NutriNet-Santé Cohort Study). Nutrients.

